# Supplementary material for: DYRK1A Overexpression in Mice Downregulates the Gonadotropic Axis and Disturbs Early Stages of Spermatogenesis
Source: Genes (Basel). 2021 Nov 16;12(11):1800. doi: 10.3390/genes12111800 (PMC8621272; doi:10.3390/genes12111800)

## Supplementary Materials

**Table S1**: Multiple-reaction monitoring transitions, declustering potentials (DP),collision energys (CE), collision cell exit potentials (CXP), quantifiers (Q), and qualifiers (q), for the 16 steroids and stable isotope internal standards.

| Compounds | Q1 mass (m/z) | Q3 mass (m/z) | DP (V) | CE (eV) | CXP (V) |
|---|---|---|---|---|---|
| Aldosterone Q | 359.2 | 189.1 | -130 | -24 | -14 |
| Aldosterone q | 359.2 | 331.2 | -130 | -23 | -22 |
| Aldosterone-d7 | 366.2 | 338.2 | -130 | -24 | -22 |
| Corticosterone Q | 347.3 | 119.1 | 130 | 40 | 14 |
| Corticosterone q | 347.3 | 121.11 | 130 | 35 | 14 |
| Corticosterone-d8 | 355.3 | 125.11 | 130 | 35 | 14 |
| Delta4-Androstenedione Q | 287.2 | 97.1 | 130 | 40 | 14 |
| Delta4-Androstenedione q | 287.2 | 109.1 | 130 | 40 | 14 |
| Delta4-Androstenedione-d7 | 294.2 | 113.1 | 130 | 40 | 14 |
| 11-Deoxycorticosterone Q | 331.3 | 109.1 | 130 | 33 | 14 |
| 11-Deoxycorticosterone q | 331.3 | 97.1 | 130 | 33 | 14 |
| 11-Deoxycorticosterone-d8 | 339.3 | 113.1 | 130 | 33 | 14 |
| Testosterone Q | 289.2 | 109.1 | 130 | 31 | 14 |
| Testosterone q | 289.2 | 97.1 | 130 | 31 | 14 |
| Testosterone-d5 | 294.3 | 113.1 | 130 | 33 | 14 |
| Progesterone Q | 315.3 | 109.1 | 130 | 35 | 14 |
| Progesterone q | 315.3 | 97.1 | 130 | 35 | 14 |
| Progesterone D9 | 324.3 | 100.1 | 130 | 35 | 14 |

**Table S2**: Primers used for real time quantitative RT-PCR of specific mRNAs.

| Gene | Forward primer | Reverse primer |
|---|---|---|
| DYRK1A | tcagtcttcaggcaccacct | tgttactcgttcccgaggat |
| Plzf | gacgcactacagggttcaca | gcttgatcatggccgagtag |
| Gfrα1 | ctcgagaaaagacacacacacc | gcctgaccatgtaaagcagtc |
| Sycp3 | ggacagcgacagctcacc | aaggtggcttcccagatttc |
| Kit-L | cagcgctgcctttccttat | ccttggttttgacaagaggatt |
| Protamine | gacagcccacaaaattccac | ctcagagcaggggacacc |
| P450scc | ggcgtgtagtgtcttagtgtcg | ggggtccacgatgtaaactg |
| StAR | aaactcacttggctgctcagta | tgcgataggacctggttgat |
| P450c17 | catcccacacaaggctaaca | cagtgcccagagattgatgat |
| 3βHSD | gaccagaaaccaaggaggaa | gcactgggcatccagaat |
| TAC3 | ctccttccttcagtctctctc | ccttttcccctcaaagctc |
| rpL19 | gggcaggcatatgggcata | ggcggtcaatcttcttggatt |
| TBP | ggggagctgtgatgtgaagt | ccaggaaataattctggctca |

**Figure S1** : Slot-blot of Dyrk1a WT and Tg mice in testis: a : Ponceau highlighting similar total protein levels; b: Dyrk1a slot-blot.

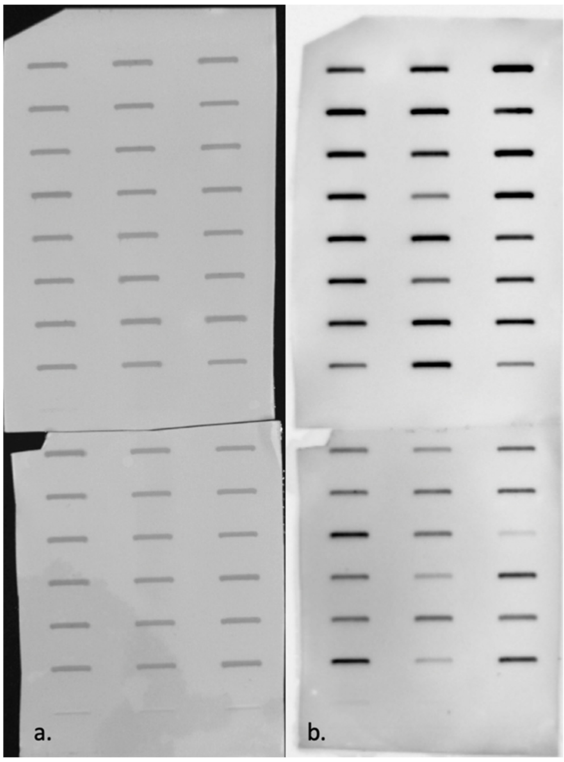

**Figure S2** : Plzf western blot in WT and Tg mice performed to assess the specificity of specific antibody. Specific anti-Plzf is found between 46 and 58 kDa.

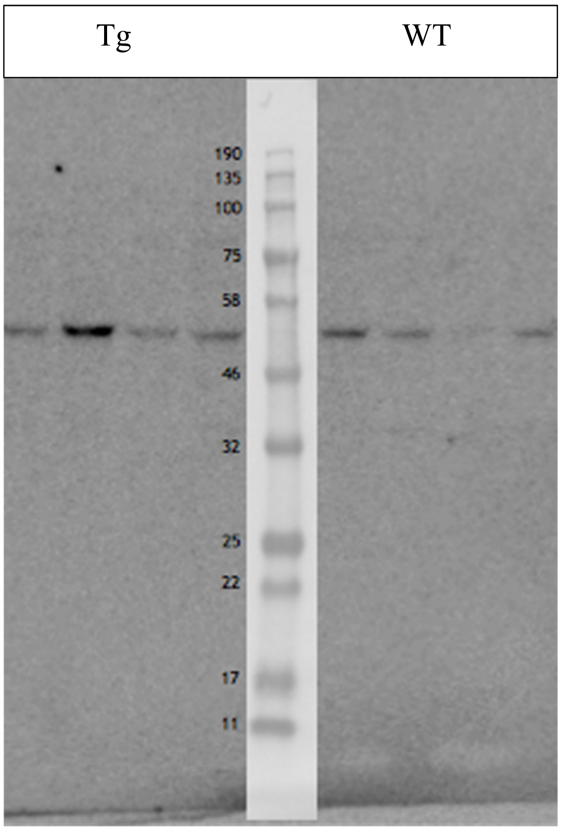

Supplement: Supplementary file 1 [file genes-12-01800-s001.zip › genes-1454690-supplementary.pdf]
